# Myalgic Encephalomyelitis/Chronic Fatigue Syndrome: Efficacy of Repeat Immunoadsorption

**DOI:** 10.3390/jcm9082443

**Published:** 2020-07-30

**Authors:** Markus Tölle, Helma Freitag, Michaela Antelmann, Jelka Hartwig, Mirjam Schuchardt, Markus van der Giet, Kai-Uwe Eckardt, Patricia Grabowski, Carmen Scheibenbogen

**Affiliations:** 1 Department of Nephrology and Medical Intensive Care, Charité—Universitätsmedizin Berlin, Corporate Member of Freie Universität Berlin, Humboldt Universität zu Berlin, and Berlin Institute of Health, 12203 Berlin, Germany; Markus.Toelle@charite.de (M.T.); Mirjam.Schuchardt@charite.de (M.S.); Markus.vanderGiet@charite.de (M.v.d.G.); Kai-Uwe.Eckardt@charite.de (K.-U.E.); 2 Institute of Medical Immunology, Charité—Universitätsmedizin Berlin, Corporate Member of Freie Universität Berlin, Humboldt Universität zu Berlin, and Berlin Institute of Health, 13353 Berlin, Germany; Helma.Freitag@charite.de (H.F.); Michaela.Antelmann@charite.de (M.A.); Jelka.Hartwig@charite.de (J.H.); Patricia.Grabowski@charite.de (P.G.); 3 Berlin-Brandenburg Center for Regenerative Therapies (BCRT), 13353 Berlin, Germany

**Keywords:** Myalgic Encephalomyelitis/Chronic Fatigue Syndrome, immunoadsorption, ß_2_ adrenoreceptor autoantibody

## Abstract

(1) Myalgic Encephalomyelitis/Chronic Fatigue Syndrome (ME/CFS) is a complex neuroimmunological disease. There is evidence for an autoimmune mechanism for ME/CFS with an infection-triggered onset and dysfunction of ß_2_-adrenoreceptor antibodies (ß_2_AR-AB). In a first proof-of-concept study, we could show that IA was effective to reduce ß_2_AR-AB and led to improvement of various symptoms. (2) Five of the ME/CFS patients who had clinical improvement following treatment with a five-day IA were retreated in the current study about two years later with a modified IA protocol. The severity of symptoms was assessed by disease specific scores during a follow-up period of 12 months. The antibodies were determined by ELISA. (3) The modified IA treatment protocol resulted in a remarkable similar clinical response. The treatment was well tolerated and 80–90% decline of total IgG and ß_2_AR-AB was achieved. Four patients showed a rapid improvement in several clinical symptoms during IA therapy, lasting for six to 12 months. One patient had no improvement. (4) We could provide further evidence that IA has clinical efficacy in patients with ME/CFS. Data from our pilot trial warrant further controlled studies in ME/CFS.

## 1. Introduction

Myalgic encephalomyelitis/chronic fatigue syndrome (ME/CFS) is a debilitating disease that is characterized by persistent fatigue and exertional intolerance with disproportionate worsening after physical or cognitive exertion. Furthermore, it is accompanied by a variety of other symptoms that are related to immunological and autonomous dysfunction [1]. With an estimated prevalence of 0.1–0.5%, ME/CFS is a frequent and chronic disease that is often triggered by an acute infection [2]. Around 2.5 million Americans suffer from ME/CFS causing an annual financial cost up to 24 billion dollars per year [3]. ME/CFS affects all races, ages, and socioeconomic groups, with women affected 2–3 times more frequently than men [4,5]. Currently, the exact pathophysiology of ME/CFS is not well understood. There is increasing evidence for an autoimmune pathomechanism in ME/CFS [6]. Autoimmunity-related risk variants in PTPN22 and CTLA4 were found to be associated with ME/CFS with infectious onset in a recent study [7]. Several studies described autoantibodies in ME/CFS, including antibodies against nuclear and membrane structures, cardiolipin, neurotransmitter receptors, and against autoantigens formed by oxidative or nitrosative damage [6,8,9,10]. Elevated autoantibodies against the muscarinic acetylcholine receptors (MAR-AB) and ß_2_-adrenoreceptor antibodies (ß_2_AR-AB) could be detected in a subgroup of patients [11,12,13]. There is first evidence for a dysfunction of adrenergic receptor antibodies in ME/CFS [14,15]. A recent study showed a correlation between ß_2_AR-AB and brain network alterations that was associated with pain [16]. Results from two clinical studies in which rituximab was used for depleting CD20+ B lymphocytes provided first evidence that B cells are involved in the pathogenesis of ME/CFS [17,18]. Approximately 60% of patients in the treatment group showed a partial or complete remission of clinical symptoms, lasting for more than six months in several patients. Interestingly, the clinical improvement was only seen after a time delay of three to four months. This indicates that the immediate depletion of CD20+ B cells has no direct effect, because CD20− antibody-producing plasma cells remain unaffected by rituximab treatment. There was a sustained reduction in ß_2_AR-AB in patients who had a clinical response to rituximab [11].

An effective treatment option for autoantibody-mediated diseases is immunoadsorption (IA). By using specific adsorbers, the plasma concentration of immunoglobulin G (IgG) can be quickly and efficiently reduced [19]. Clinical symptoms of various autoimmune diseases that are associated with autoantibodies, including dilative cardiomyopathy, therapy refractive lupus erythematosus, and various neurological diseases, could be improved quickly by IA [20,21,22]. Recently, we described a proof-of-concept prospective observational IA study in ten patients with ME/CFS, in whom ß_2_AR-AB were elevated [23]. Five cycles of IA were conducted on days one–three and six–seven. In nine patients, the ß_2_AR-AB decreased rapidly during IA treatment and it was still significantly lower than the pretreatment level after six months. The frequency of memory B cells significantly decreased, whereas the frequency of plasma cells increased after a five-day IA cycle. A rapid improvement of symptoms was reported by 70% of patients already during IA. Three of these patients had long lasting improvement for more than 12 months and four patients had short improvement. However, two patients had a marked worsening of symptoms during IA and could not receive the fifth IA.

Here, we present data of a conformational trial. Patients who had responded to the first IA (IA1) were retreated with a modified IA protocol (IA2) about two years later. Here, IA cycles were given with longer intervals.

## 2. Materials and Methods

### 2.1. Patients

**Figure 1:**
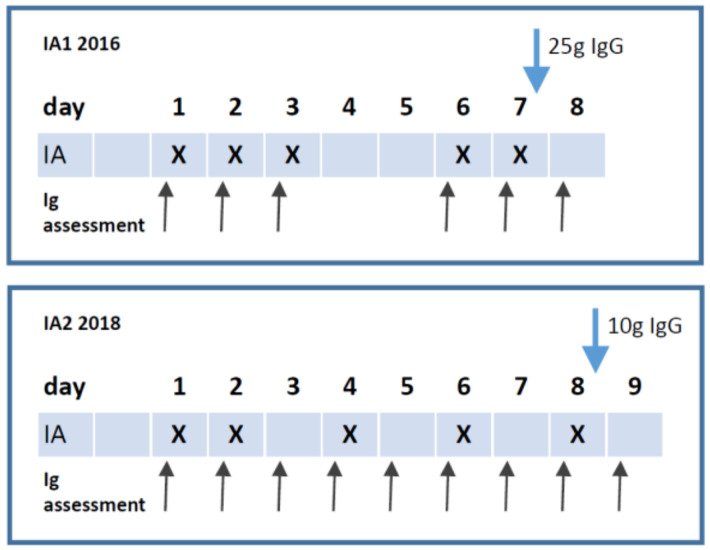
Treatment protocols of both studies. X indicates the point of time when immunoadsorption (IA) was conducted, black arrows when blood samples were collected, and blue arrow for immunoglobulin G (IgG) supplementation.

For the conformational trial, we adjusted the treatment protocol (IA2, see Figure 1). Five of the ten participants of our first study in 2016 were included in the current study. These patients had a transient or long-lasting improvement of clinical symptoms after the first IA therapy. Detailed inclusion criteria were described before [23]. In short, all of the patients fulfilled the Canadian Consensus Criteria [1], had increased ß_2_AR-AB levels, and an infection-triggered disease onset.

### 2.2. Study Protocol

During the IA1 study, we learned that repeated IA can worsen fatigue. Four patients had worsening of fatigue towards the end of treatment despite improvement of other symptoms. To improve the tolerability of the treatment, we extended the treatment period and reduced the dose of IgG replacement. IA was performed at day one, two, four, six, and eight (Figure 1) using Globaffin^®^ columns (Fresenius, Bad Homburg, Germany), a broadband-immunoadsorber containing synthetic peptide-GAM^®^ as ligand capable of binding IgG and immune complexes independent from their antigen specificity and, thus, useful for the removal of autoantibodies. Patients received 10 g polyclonal immunoglobulin substitution intravenously (Octagam, Octapharma, Langenfeld, Germany or Gammunex, Grifols, Frankfurt/M., Germany) to restore IgG plasma levels after the final IA session. The study was approved by the Ethics Committee of Charité - Universitätsmedizin Berlin (project code: EA2/063/15 from 10 September, 2018) in accordance with the 1964 Declaration of Helsinki and its later amendments. All of the patients gave written informed consent.

### 2.3. Assessment of Autoantibodies, Ig, Albumin and Fibrinogen

ß_1_AR-/ß_2_AR-AB and M3AR-/M4AR-AB were determined using ELISA technology by CellTrend GmbH (Luckenwalde, Germany), as in our previous study [23]. Total serum IgG, IgA, IgM, albumin, and fibrinogen were determined at the Charité diagnostics laboratory (Labor Berlin GmbH, Berlin, Germany).

### 2.4. Symptom Assessment by Scores

We assessed the presence and severity of symptoms, as described in our previous study [23]. In short, the patients quantified the severity of symptoms of the Canadian consensus criteria using a questionnaire that was developed by Fluge et al. [17,18]. After the determination of a baseline value, patients stated the improvement or worsening of symptoms (0–3: worsening; 3: no change from baseline; 3–6: improvement). The patients filled this questionnaire daily during treatment and monthly during follow-up. Furthermore, patients evaluated fatigue and cognitive impairment monthly using FACT-F questionnaire [24].

### 2.5. Statistical Analysis

We conducted statistical data analyses using GraphPad Prism version 6.0 software similarly to the first proof-of-concept study [23]. In short, we used nonparametric statistical methods, median and interquartile range (IQR) for continuous variables. For univariate comparisons, we used Wilcoxon matched-pairs signed-rank test and Mann–Whitney-U test or Fisher’s exact test for independent groups. A two-tailed *p*-value of <0.05 was considered to be statistically significant.

## 3. Results

### 3.1. Patient Characteristics and IA Treatment

All of the patients had an infection-triggered onset of ME/CFS and all showed elevated levels of ß_1_/ß_2_-AR-AB and M3/M4-AR-AB (except patient 8 only ß_2_). Bell disability scale indicating the severity of disease ranged from 30–75 (median of 45) and the median was significantly higher than before the first IA study in 2016 (median of 30, range 10–50, *p* = 0.01, Figure S1) corresponding to an increase of the ability to perform desk work from 2–3 h to 4 h daily [25]. Table 1 shows patient characteristics. The intervals between the cycles were extended in the present protocol in order to improve the tolerability of the treatment. Five cycles of IA were conducted within eight days (day 1, 2, 4, 6, and 8). A citrate-based anticoagulation was used. We observed a drop of fibrinogen and albumin levels in a similar extent compared to the first study (Figure S2). No albumin substitution was necessary and there was no bleeding episode. Immediately after the fifth IA, all apatients received 10 g IgG intravenously, as compared to 25 g in the first proof-of-concept study.

### 3.2. Course of IgG and Autoantibodies

**Figure 2:**
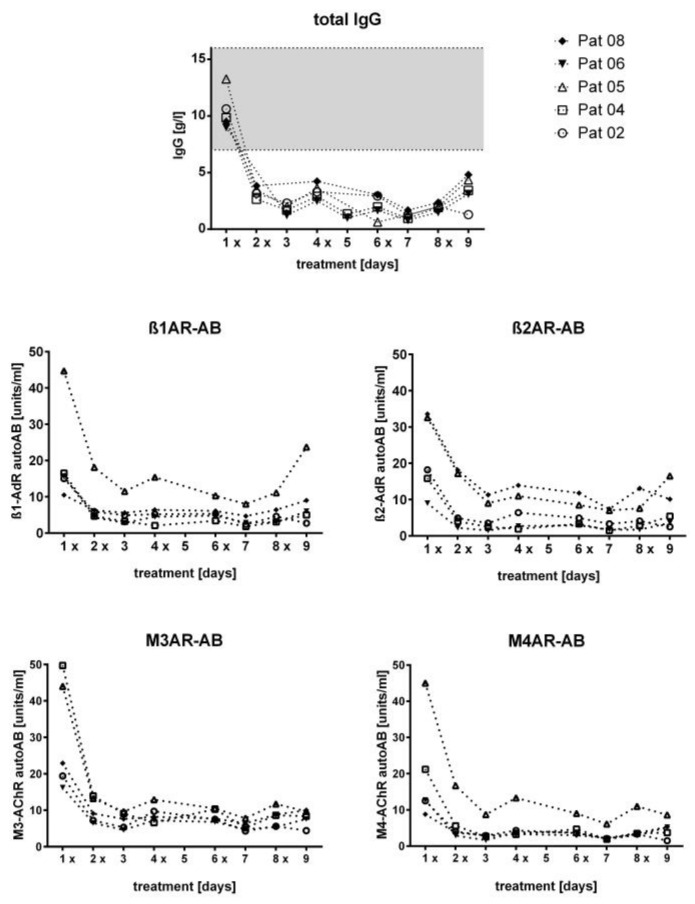
Absolute IgG and autoantibody levels during treatment. Total and ß_1_, ß_2_, M3, and M4 IgG in the serum before and during IA. X indicates the point of time when IA was conducted. Gray area indicates reference range of serum levels.

**Figure 3:**
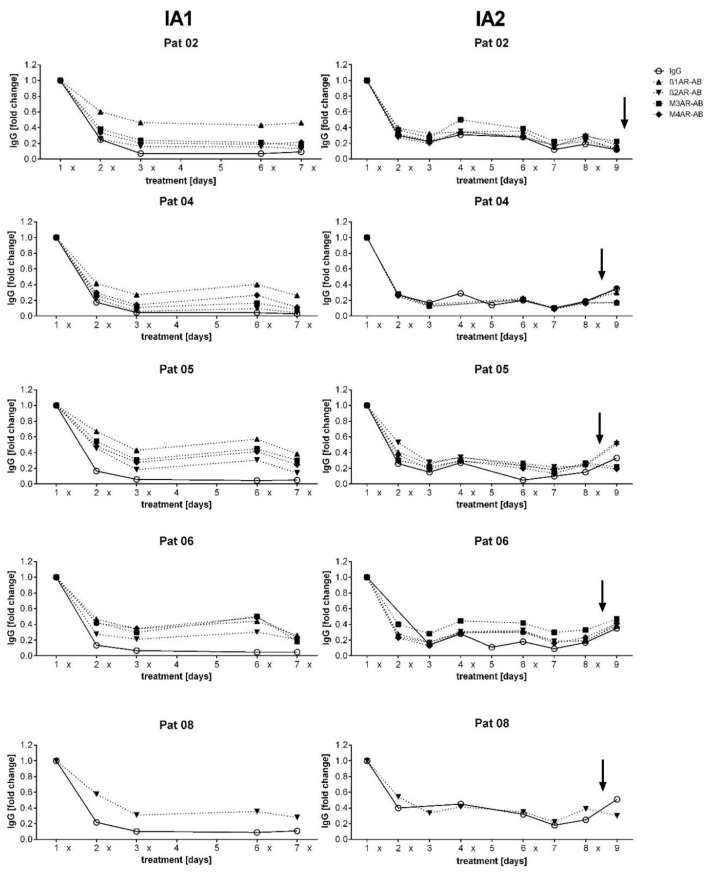
Relative IgG and autoantibody levels during treatment. Relative changes of total IgG and ß_1_, ß_2_, M3, and M4 autoantibody concentration in the serum before and during first IA 2016 (left) and second IA (right). The daily levels are depicted as x-fold change to day 1 level for each single patient. X indicates the single IA, arrow when patients received 10 g IgG i.v.

Before IA treatment, the total IgG levels were within the normal range in all patients (median 9.85 g/L, range 8.99 to 13.26 g/L). As expected, the absolute IgG levels were strongly reduced already after the first IA with a minimum IgG level after the fourth IA (median 0.95, range 0.64 to 1.68 g/L). The absolute level of autoantibodies decreased in a comparable way, as shown in Figure 2. Figure 3 shows the relative decrease in IgG and autoantibody concentrations in each individual patient. The IgA and IgM levels did not change significantly (Figure S3).

### 3.3. Clinical Course

**Figure 4:**
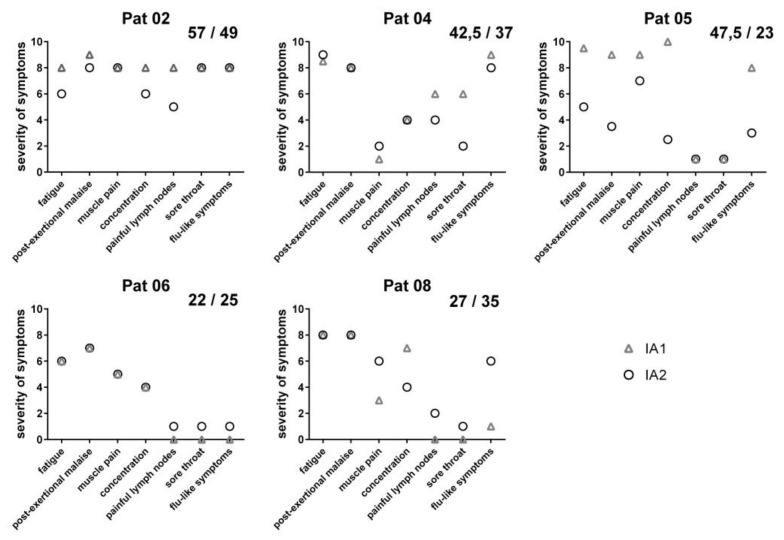
Patients condition before first and second treatment. Symptom scores before first IA (triangle) and second IA (circle). Symptoms are indicated as 0 (absent) to 10 (most severe). Sum of each patient is displayed in the upper right corner (left: IA1, right: IA2).

Before IA1, all of the patients suffered from severe exhaustion and post-exertional malaise grade 6–10 (0 none, 10 most severe symptoms) and from concentration impairment grade 4–10, muscle pain (*n* = 4), and immune-associated symptoms of sore throat, flu-like symptoms, and painful lymph nodes (Figure 4). Two years later, before the IA2, the total score of symptoms had improved in three of the five patients (patient 2, 4, and 5), was similar in patient 6, and slightly worse in patient 8 (shown in Figure 4). However, the Bell score was improved in patient 8 from 10 to 30 as she could not walk before the first IA due to marked muscle fatigue, which had considerably improved following the IA1.

**Figure 5:**
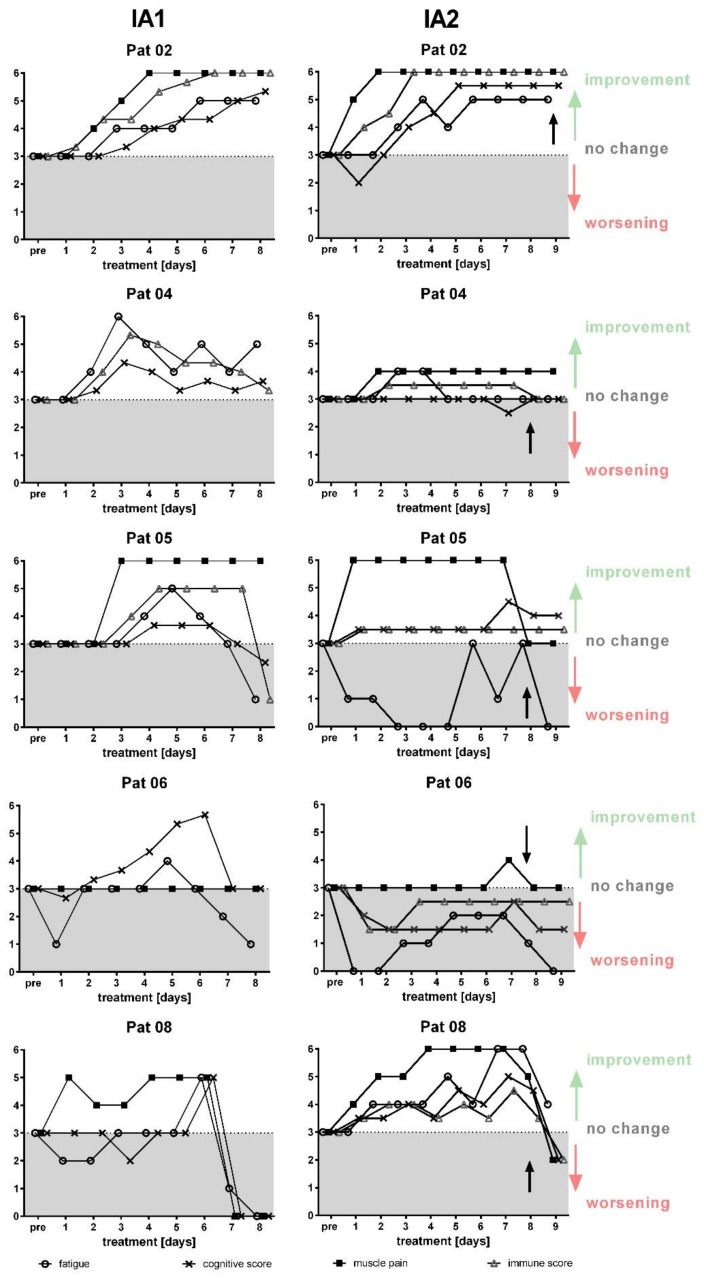
Development of symptoms during IA. Symptom scores for fatigue, cognitive score, muscle pain and immune score during IA1 (left) and IA2 (right) are shown for each patient (3 unchanged, 4 slight, 5 marked improvement, 6 complete disappearance, 2 slight increase, 1 marked increase). The line indicates level 3 for unchanged symptoms.

**Figure 6:**
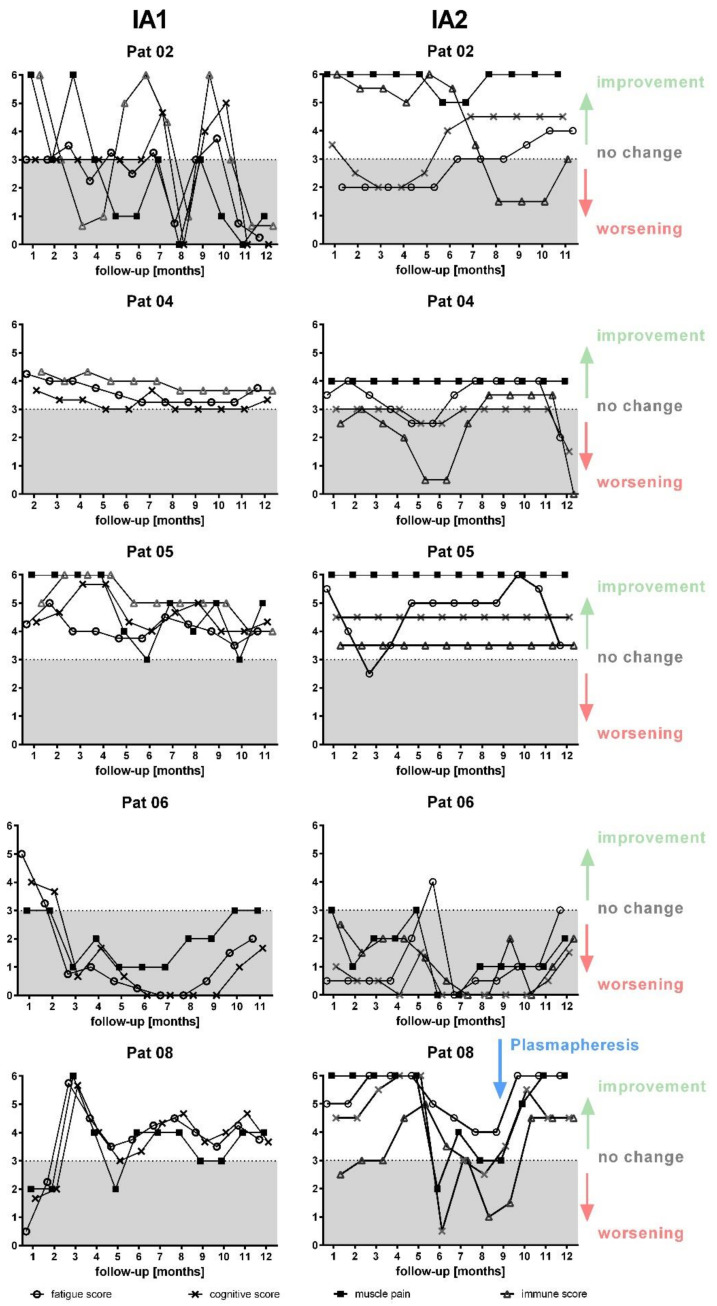
Development of symptoms during 12 months follow-up. Symptom scores for fatigue, cognitive score, muscle pain and immune score during first IA (left) and second IA (right) are shown for each patient (3 unchanged, 4 slight, 5 marked improvement, 6 complete disappearance, 2 slight increase, 1 marked increase). The line indicates level 3 for unchanged symptoms.

The assessment of symptoms was performed daily during the IA2 (Figure 5) and afterwards monthly until month 12 (Figure 6). Interestingly, the course of symptoms was similar in IA2 as compared to IA1. Patients 2 and 8 (Figure 5) showed rapid improvement of all symptoms during IA2. In patient 4, muscle pain and immune symptoms improved. In patient 5, muscle pain disappeared during IA and cognitive and immune symptoms slightly improved, but fatigue worsened. Patient 6, who had a short-term improvement of cognition during IA1, had no improvement during the IA2. Patients 5, 6, and 8 had a transient worsening of symptoms after IgG infusion at day eight.

The course of symptoms during the 12 months after IA2 was again similar to the IA1 in all patients (Figure 6). Patient 2, 4, and 5 again showed a sustained improvement in symptoms for ten to 12 months, although with some fluctuations. Patient 8 had marked improvement for six months. When patient 8 worsened at month six a further IA treatment was offered, but she preferred to receive plasmapheresis on month 9 in her local hospital, which again almost completely resolved the symptoms. Patient 6, who had a two months improvement during IA1 with consecutive worsening, experienced an immediate worsening under IA2.

**Figure 7:**
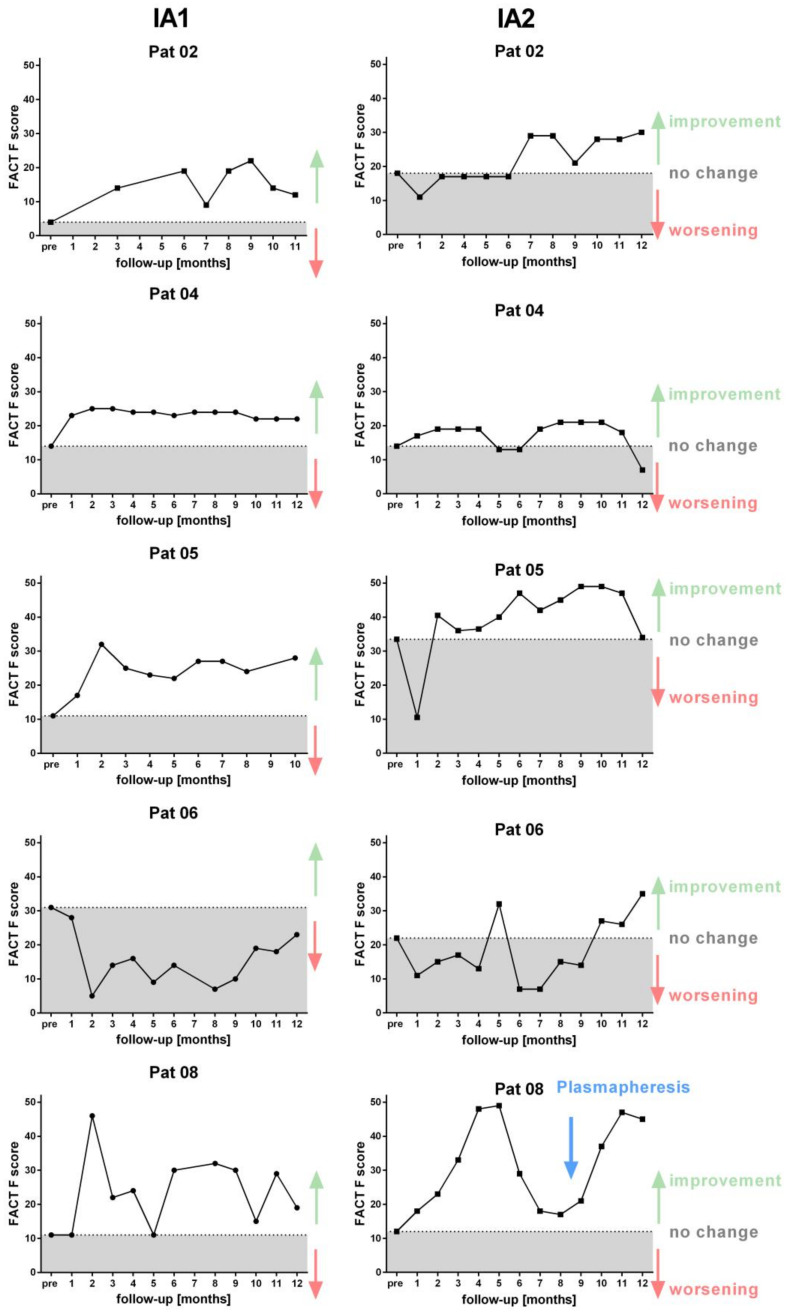
FACT-F score follow-up. Score of FACT-F questionnaire assessing severity of fatigue before and up to 12 months after first IA (left) in comparison to second IA (right) for each patient. Scoring of FACT-F fatigue questionnaire ranges from 0 (strongly fatigued) to a maximum of 52 (without fatigue). Dotted line indicates the individual pretreatment score before IA.

Further patients filled in FACT-F questionnaire monthly, assessing the severity of fatigue showing a similar course to IA1. A strong improvement of fatigue was reported by patients 5 and 8, while patient 2 and 4 only had a slight improvement of fatigue in accordance to the fatigue that was reported in the symptom score (Figure 6 and Figure 7).

Regarding tolerability, three of the five patients had a worsening of fatigue from day 6 on during IA1, but two patients had marked worsening of fatigue also during IA2.

## 4. Discussion

In our first proof-of-concept study in ten patients with infection-triggered ME/CFS, we observed that IA caused a rapid decrease in ß_2_AR-AB levels in nine of ten patients. Moreover, an improvement in clinical symptoms could be achieved in seven of the patients, which lasted, in three patients, for more than 12 months [23]. Furthermore, the ME/CFS patients frequently suffer from endothelial dysfunction, which was also improved following IA [26]. These results provided first evidence that IA may be a therapeutic option in ME/CFS patients and the rapid relief from clinical symptoms may be explained by the removal of autoantibodies. We also found a decrease of memory B cells following IA1 and significantly lower ß_2_AR-AB after six months, suggesting that IA may have an effect on autoreactive memory B cells as well. Repeated IA were shown in other autoimmune diseases to enhance efficacy or induce a second remission [19,20,21].

Therefore, we conducted a conformational study in five patients with a clinical response in the first study and retreated them with second IA. Remarkably, disease severity was still improved before IA2 when compared to before IA1 two years earlier, with a higher Bell score in all five patients. During IA1, two patients had considerable worsening of all ME/CFS symptoms, which may be attributed to their enhanced susceptibility to stress and changes in the water and electrolyte balance. In addition, four patients had worsening of fatigue during IA1. Despite longer intervals between the cycles during IA2, two patients, however, had again marked worsening of fatigue. When compared to the IA1 protocol, the IA2 protocol resulted in a comparable decrease in IgG and autoantibody concentrations.

As in the IA1 study, the clinical symptoms that were associated with ME/CFS were assessed with a questionnaire quantifying the most important symptoms of the CCC [1,17] The descriptions of subjective self-reported symptom correlated well with the objective activity tracking of steps per day in IA1 [23]. Similar to IA1, we could observe an improvement of several clinical symptoms during IA in four of five patients lasting with some fluctuations for 6–12 months. The patient who experienced marked worsening also only had a moderate and transient improvement in symptoms after IA1.

Of interest is that one patient received a plasmapheresis nine months after IA2, which again almost completely resolved the symptoms. Plasmapheresis constitutes another possibility to eliminate pathogenic antibodies by exchange of patient with donor plasma, but its efficacy has not been assessed in ME/CFS to our knowledge. In other diseases, it was shown that IA and plasmapheresis both resulted in comparable clinical efficacy [27].

Our observations are in line with the clinical results of IA in neuro-immunological diseases or dilative cardiomyopathy [27,28]. Dilative cardiomyopathy is often associated with ß_1_AR-AB, which could be effectively reduced by IA leading to a long-term improvement in the clinical outcome [28]. Clinical features of a refractory lupus erythematosus, like proteinuria, SLEDAI (activity index for systemic lupus erythematosus disease) and autoantibody concentrations, could be successfully improved and stabilized at levels that meet the criteria of remission in the long term by the regular use of an IA for up to ten years [20].

In our first study, a sustained decrease of ß_2_ IgG was observed at month six, which could also be shown in responder to the rituximab therapy [11]. The long-term decline of autoantibody concentrations may be explained due to enhanced B cell differentiation with a higher apoptosis rate of autoreactive B cells and consecutive loss of short living autoreactive plasma cells [11]. This hypothesis would correspond to the observed decrease in ß_2_AR-AB six months after IA1, when the levels of total IgG and tetanus and pneumococcal IgG corresponded to the pretreatment ones [11]. However, the effect of adrenergic stimulation on immune cells is complex and does not directly correlate with levels of ß_2_AR-AB [14]. ß_2_AR-AB belong to a network of natural antibodies against G-protein coupled receptors, which has been described to be dysregulated in various autoimmune diseases [29]. In our recent study, we found that ß_2_AR-AB activate the ß_2_AR. The ß_2_AR activation by IgG was attenuated in ME/CFS patients, which could explain many symptoms of ME/CFS [14]. As IA removes total IgG, we have, however, no direct evidence from our study that the removal of ß_2_AR itself leads to improvement. Several other autoantibodies were reported in ME/CFS [6].

In this study, the patients received a single IgG infusion at the end of IA protocol in order to partially restore the strong IgG depletion. IgG treatment is also effective in autoantibody-mediated autoimmune diseases [30]. Doses applied for treatment of autoimmune disease are usually above 1.0 g/kg body weight. In the current study, an approximately 10-fold lower and single dose of IgG was applied, so that the treatment effect due to the IgG administration seems unlikely.

Limitations of the current trial are the small number of patients and the lack of a placebo control group. One aspect of this study was to investigate the reproducibility of the effect of IA on clinical symptoms in a subset of patients from the first study. In all patients, the effect of IA treatment could be reproduced. Although some placebo-treated patients showed an improvement in randomized placebo-controlled trials (RCT) in ME/CFS [18,31], the reproducibility and the long lasting symptom improvement associated with a significantly decreased frequency of memory B cells, increased frequency of plasma cells, and lower autoantibody concentration six month after IA1 rather speaks against an unspecific effect. Further, although being considered unlikely, IgG replacement at the end of IA may have an immunomodulatory effect. Therefore, we have intentionally kept the IgG dose in the current study lower to minimize this potential effect.

Our study was designed to get further evidence for efficacy and tolerability prior to performing a consecutive RCT. We have evidence for similar clinical and immunological efficacy of this schedule.

## 5. Conclusions

In summary, the current study provides further evidence that IA is effective in ME/CFS. This result warrants further studies of repeat IA therapy to maintain clinical remission, as shown for other autoimmune diseases [20]. Another option is to include IA in a therapy algorithm as initial therapy for autoantibody-positive ME/CFS patients in order to achieve rapid symptom relief and, therefore, shorten the known time latency of four months or longer until the onset of efficacy of B cell-targeting therapies.

## Figures and Tables

**Table 1:** Patients’ characteristics.

Patient No. from 2016 Study	Gender	Age	ME/CFS Onset	Disease Severity Bell Score before IA1	Disease Severity Bell Score before IA2
2	f	60	2011	30	45
4	m	50	2000	20	35
5	f	37	2012	40	75
6	f	32	2002	50	60
8	f	32	2005	10	30

## Supplementary Materials

The following are available online at https://www.mdpi.com/2077-0383/9/8/2443/s1, Figure S1: Initial Bell score., Figure S2: Albumin and fibrinogen serum concentrations., Figure S3: IgA and IgM serum concentrations.
