# Supplementary material for: Myalgic Encephalomyelitis/Chronic Fatigue Syndrome: Efficacy of Repeat Immunoadsorption
Source: J Clin Med. 2020 Jul 30;9(8):2443. doi: 10.3390/jcm9082443 (PMC7465279; doi:10.3390/jcm9082443)
Supplement: Supplementary file 1 [file jcm-09-02443-s001.pdf]

Journal of Clinical Medicine

MDPI

Article

Myalgic Encephalomyelitis/Chronic Fatigue Syndrome: efficacy of repeat immunoadsorption

Supplements:

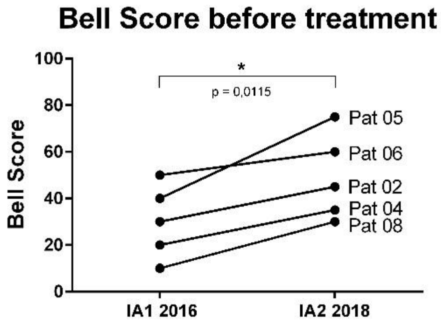

Figure S1. Initial Bell score. Bell Score of each patient before first IA (2016) and second IA (2018). Bell Score significantly increased since first IA. Scoring of Bell questionnaire ranges from 100 (no restrictions of activity) to 0 (no discrete cognitive or physical action possible). Paired t-test, p value < 0,05 was considered as significant.

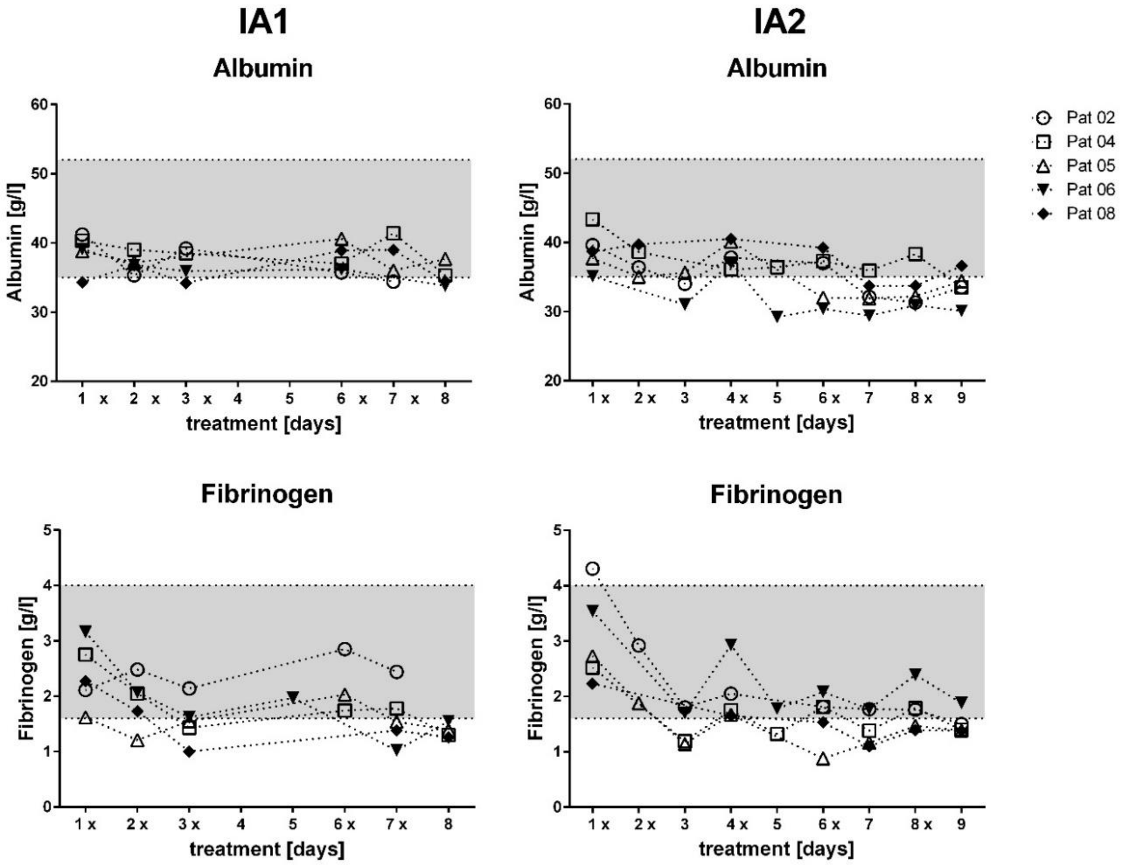

Figure S2. Albumin and fibrinogen serum concentrations. Absolute albumin and fibrinogen levels during treatment of first IA (left) and second IA (right). X indicates the point of time when IA was conducted. Gray area indicates reference range of serum levels.

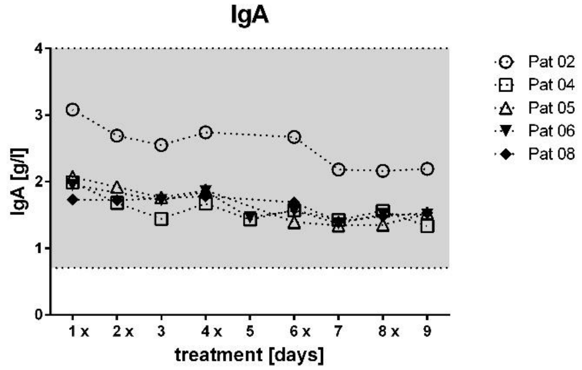

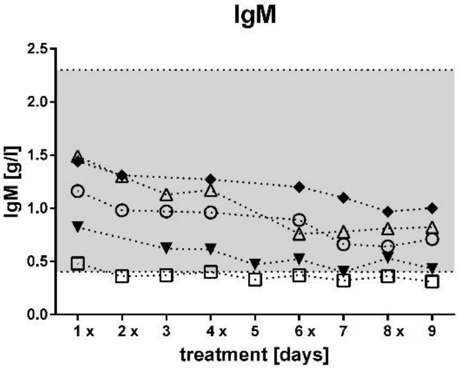

Figure S3. IgA and IgM serum concentrations. Course of IgA and IgM levels during treatment. Levels remain mostly unaffected by IA. X indicates the point of time when IA was conducted. Gray area indicates reference range of serum levels.
